# SEGMENTAL NEUROFIBROMATOSIS: A REPORT OF 3 CASES

**DOI:** 10.4103/0019-5154.60366

**Published:** 2010

**Authors:** Sushma Kashinath Gabhane, Mrunmayi Nishikant Kotwal, Sudhakar K Bobhate

**Affiliations:** *From the Department of Pathology, Mahatma Gandhi Institute of Medical Sciences, Sevagram, Wardha, India*; 1 *From the Department of Government Medical College, Nagpur, India.*

**Keywords:** *Segmental neurofibromatosis*, *neurofibromatosis 1*, *café-au-lait spots*, *neurofibroma*

## Abstract

Neurofibromatosis is a genetic disorder of neural crest-derived cells that primarily affect growth of neural tissues. It is broadly divided into three categories: (a) von Recklinghausen's neurofibromatosis or NF-1, (b) bilateral acoustic neuroma (NF-2), and (c) all other neurofibromatoses, including alternate or atypical forms of the disease. The patients with generalized form of NF1 are characterized by multiple café-au-lait spots and neurofibromas and diagnosed easily. But when an individual has small number of lesions in a limited region of the body it could be neglected by the patient or not be recognized by the clinicians as a segmental form of neurofibromatosis. We describe three cases of segmental neurofibromatosis (SNF). These cases have been classified as segmental NF according to Riccardi's definition of SNF and classification of neurofibromatosis. Segmental form of NF may evolve into a complete form over time. Also, this disorder may be transmitted to the offspring's of these individuals. Hence genetic counseling of these individuals must include these facts.

## Introduction

Neurofibromatoses are a set of inherited disorders designated as neurofibromatosis type 1 (NF1), neurofibromatosis type 2 (NF2), and schwannomatosis. They tend to result in the development of benign tumors of nerve sheath. The three clinical entities are distinguished by specific clinical features and are due to mutations in distinct genes. Neurofibromatosis type 1 is the most common of these disorders, affecting approximately 1 in 3500 individuals worldwide and with nearly 100% penetrance of the disease.[1,2]

Segmental neurofibromatosis (SNF) was first described by Crowe *et al*.[3] in 1956 and the authors termed it *Sectorial neurofibromatosis*. In 1977, Miller and Sparkes[4] renamed this term as SNF. Riccardi[5] included SNF in his classification of neurofibromatosis (NF) as type V. The clinical features of SNF were also established by him as: Café-au-lait spots and/or neurofibromas in a single unilateral segment of the body, with no crossing of the median line, no family history, and no systemic involvement. Roth *et al*.[2] has further subdivided the SNFs into four subtypes: True segmental, localized with deep involvement, hereditary, and bilateral.

We describe three patients who showed segmental expression of the disease and review the previous literature.

## Case Reports

### Case 1

A 42-year old man presented with multiple small nodules in the ulnar aspect of right forearm in the outpatient department of surgery. The patient was also noted to have two café-au-lait macules of 1.2 cm and 1.6 cm diameter on the same forearm, present since childhood. The nodules had gradually developed and increased in size over a period of 15-20 years, according to him. There was no history of similar lesions in any of the family members. There was no any significant past medical history.

**Figure 1:**
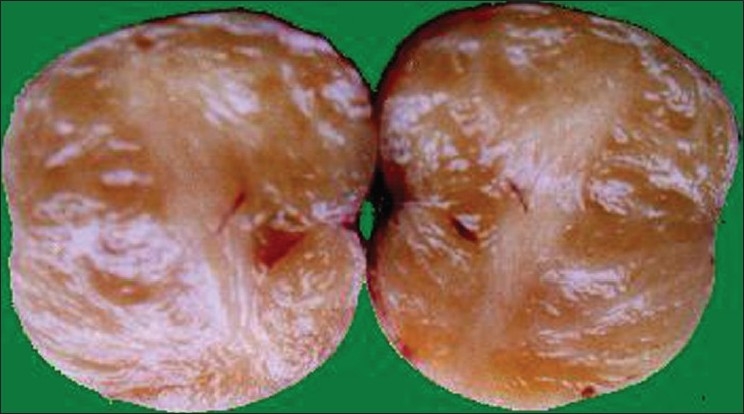
Gross photograph of a neurofibroma from case 1 showing homogeneous gray-tan cut surface

On clinical examination, four skin colored, firm, painless nodules ranging in size from 5 mm to 1.6 cm in diameter were seen to be distributed on flexor aspect of the right forearm near the ulnar border. No similar lesions were found elsewhere. Axillary freckling, plexiform neurofibromas, Lisch nodules or any skeletal deformities were absent. Excisional biopsy of two nodules was done; [Figure 1]. On histopathology, both of them revealed a nonencapsulated but well circumscribed spindle cell neoplasm, with cells arranged in fascicles, spindle to wavy, with buckled nuclei, indistinct cytoplasm and myxoid background. Nuclear pleomorphism, mitosis and necrosis were not seen. Hence the diagnosis of a usual type of neurofibroma was entertained.

### Case 2

A 34-year-old female presented with a history of multiple small nodules, which first appeared on the left lateral side of her chest wall as a single nodule at the age of 16 years. The patient did not seek any investigations or treatment as the lesion was very small and painless. Since then, she noted gradually increasing numbers of these lesions in the same area. She had no family history of neurofibromatosis.

**Figure 2:**
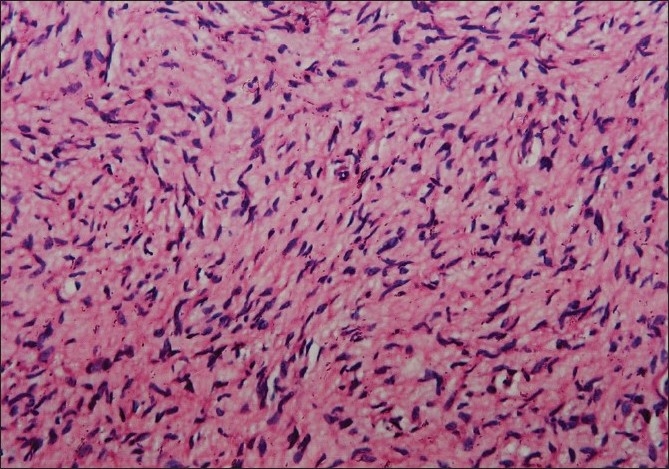
Photomicrograph showing wavy, buckled nuclei of Schwann cells (H and E stain, ×200)

Physical examination disclosed three soft to firm tumors of 1 cm to 2.5 cm diameter on the left lateral chest wall just below the axilla. Axillary freckling was also noted on the same side. There were no café-au-lait spots, Lisch nodules by slit lamp examination, or any other neurofibromas. Routine hematological and biochemical investigations done prior to excision of tumors were within normal limits. Histopathological examination of tumors showed a usual type neurofibroma [Figure 2].

### Case 3

A 17-year-old male disclosed a seven to eight year history of asymptomatic nodules on left lower back and abdomen. The lesions did not cross the midline. Also, since infancy, the patient had hyperpigmented macules on the left lower aspect of back. These lesions also stayed on one side of midline. His family history was not significant.

**Figure 3:**
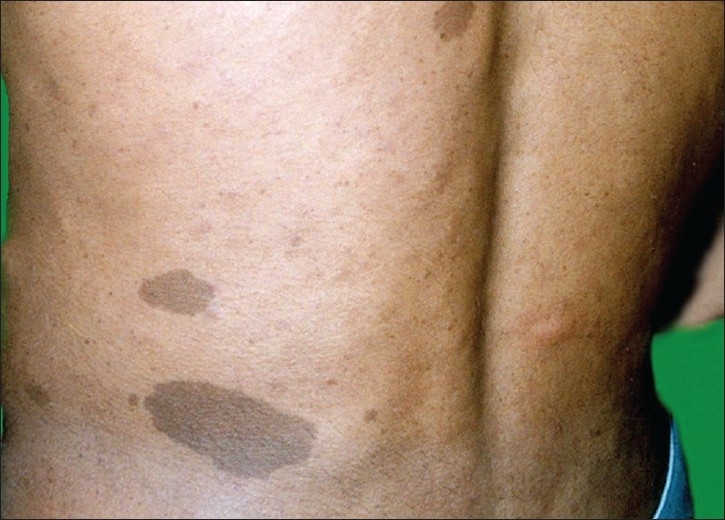
Clinical photograph of a patient in case 3 showing three cafe.au.lait spots on the left side of lower back along with freckling

**Figure 4:**
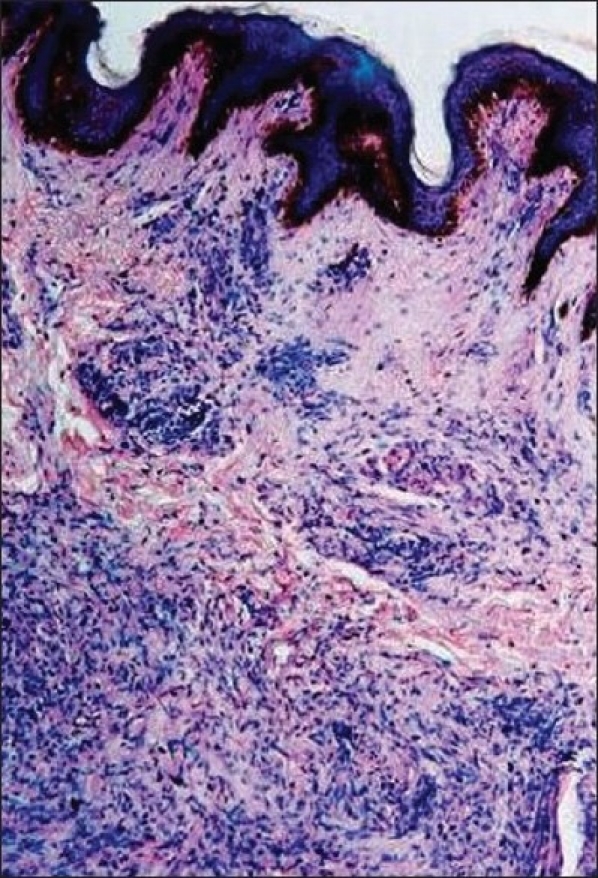
Photomicrograph of a café-au-lait spot showing increased melanin pigment in basal layer of epidermis and a underlying neurofibroma in the dermis (H and E stain, ×40)

Physical examination showed three café-au-lait spots of 1.5 cm to 2.5 cm diameter [Figure 3] and more than 10 soft, small, skin colored nodules of variable size in a dermatomal distribution on the left side of the lower back and abdomen along with intertriginous freckling. No Lisch nodules or skeletal deformities were detected. A biopsy specimen of one of the nodules showed a circumscribed, nonencapsulated neurofibroma with overlying café-au-lait spot [Figure 4].

## Discussion

These three patients showed relatively localized manifestations of neurofibromatosis limited to dermatomal distribution of a particular nerve root and not crossing the midline. Also, all these patients had negative family history for neurofibromatosis. Hence, we believe that all these patients have a form of neurofibromatosis which can be classified as SNF according to Riccardi's classification.

Neurofibromatosis is extremely variable in its presentation. Although generalized expression of the disease develops in most cases (90%), some cases present with localized neurofibromas or café-au-lait spots.[2,5] This was first described by Crowe *et al*. as sectorial NF. They proposed that somatic mutation gives rise to neurofibromatosis limited to a distinct sector of the body, with no genetic transmission of the trait.[3]

Recognizing the heterogeneity of the disease, Riccardi[5] suggested a useful system for classifying NF into eight subtypes (NF-I to NF-VIII). One of these types (NF-V) is segmental NF, defined by limitation of café-au-lait spots and neurofibromas to a given region of the body. Riccardi believed that a postzygotic somatic mutation, in primitive neural crest cells, is the most likely cause for development and thus the lesion should be strictly non-inherited. He defined SNF as café-au-lait macules or neurofibromas in a single, unilateral segment of the body, with no crossing of midline, no family history, and no systemic involvement.[5] Cases that did not conform to these criteria prompted further classification into the following subtypes: True segmental, localized with deep involvement, hereditary, and bilateral.[2]

Weiss *et al*.[6] modified the classification of NF1 as there appeared to be variant forms in which the features are atypical or incomplete. These are i) “Classic” NF1 ii) Whole gene deletion phenotype NF 1, and iii) Alternate forms of NF1 (i.e. with incomplete/atypical features). Segmental NF1 is included in the third category as a localized form of neurofibromatosis.

SNF is a rare disorder with its prevalence estimated between 0.0014 and 0.002%.[7] It is an example of mosaicism in which localized disease results from a postzygotic NF1 gene mutation located on the proximal long arm of chromosome 17. Gene mutation can arise in both somatic and gonadal cell lines. Gonadal mosaicism is thought to be responsible for reports of patients with localized disease having children with generalized NF1.[7,8]

Ingordo *et al*.[9] observed a group of 56,183 young men between the age of 17 and 18 years representing a population homogeneous with reference to age, sex, race, and country of origin. In this group 11 cases of NF, with relative frequency of 0.020%, have been found. Instead, during the same period, only one case of SNF has been observed (relative frequency 0.0018%). From these observations, the authors concluded that SNFs are probably not underdiagnosed but are 10 times more infrequent than other forms of NF.

The recent increase in the reported cases of SNF shows the rising interest in this variant of the syndrome and indicates a justifiable doubt about the rarity and underdiagnosis of SNF. Many times, the clinical picture of SNF can be neglected by the patient and passed unnoticed by the clinicians because of absence of symptoms.

Clinically, patients may be divided into four groups: (i) with only pigmentary changes, (ii) with only neurofibromas, (iii) with both pigmentary changes and neurofibromas, and (iv) with isolated plexiform neurofibromas. Lesions are usually unilateral although there are reports of bilateral segmental NF.[7] Clinical disease develops along the same time course as generalized NF with pigmentary changes and neurofibromas, and plexiform neurofibromas developing in childhood and neurofibromas developing in adulthood.[7] Most commonly, patients present with only neurofibromas. The neurofibromas are mostly asymptomatic and range in size from 0.1 cm to several centimeters in diameter. They tend to arise in dermatomal distribution, most commonly cervical, followed by thoracic, lumbar, and sacral region.[10] Pigmentary changes include, café-au-lait macules and axillary freckling, with the former being more common.[10] In most cases, pigmentary changes follow the lines of Blaschko. Some patients of SNF have had complications of NF1, which have included learning difficulties, optic pathway gliomas, and pseudoarthrosis. Those with learning difficulties tended to have large areas of cutaneous involvement. Lisch nodules are rarely seen in segmental NF.[7]

In view of these features, what approach should be adopted for the patient who presents with localized lesions of NF? First, a through physical examination should include a search for neurofibromas, Lisch nodules, or café-au-lait spots elsewhere. If the lesions are found bilaterally, systemically, or in family members, then the patient does not fit into the strict definition of NF-V (i.e. segmental NF). Even if the patient adheres to the definition set forth by Riccardi, in time he may develop lesions systematically or in a distinct site, and the disease would have to be reclassified as a different subgroup of segmental NF. Also, patients with SNF have been found to transmit NF to their children.[7]

In the light of this risk, the clinical diagnosis of SNF is tentative, and more intensive initial evaluation as well as regular follow up examinations should be considered.

There are no specific guidelines regarding management for segmental NF. Patient should be informed that they do not have generalized NF 1 and their risk of disease associated complications is low.[7]
